# Chemical Composition, and Antioxidant and Antimicrobial Properties of *Monarda didyma* L.’s Essential Oils and Hydrosols

**DOI:** 10.3390/molecules31132252

**Published:** 2026-06-26

**Authors:** Patrycja Cichosz, Magdalena Walasek-Janusz, Agnieszka Grzegorczyk, Rafał Papliński, Piotr Kiczorowski, Renata Nurzyńska-Wierdak

**Affiliations:** 1 Department of Vegetable and Herb Crops, Faculty of Horticulture and Landscape Architecture, University of Life Sciences in Lublin, 54 Doświadczalna Street, 20-280 Lublin, Poland; patrycja.cichosz@up.lublin.pl (P.C.); magdalena.walasek@up.lublin.pl (M.W.-J.); rafal.paplinski@up.lublin.pl (R.P.); piotr.kiczorowski@up.lublin.pl (P.K.); 2 Chair and Department of Pharmaceutical Microbiology, Faculty of Pharmacy, Medical University of Lublin, 1 Chodzki Street, 20-093 Lublin, Poland; agnieszka.grzegorczyk@umlub.edu.pl

**Keywords:** medicinal plants, distillation, secondary metabolites, polyphenols, thymol

## Abstract

Aromatic medicinal plants are a constant focus of interest for scientists and producers. One example is *Monarda didyma* L., an aromatic perennial with proven health benefits. During the distillation process, hydrophobic (essential oils) and hydrophilic (hydrosols) fractions were obtained from the leaves, flowers and aerial parts (herb) of bee balm (*M. didyma*) in our study. The highest yield of essential oil (mL·100 g^−1^ DM) was obtained from the flowers, whilst the highest yield of hydrosol was obtained from the leaves (42.46 mL·100 g^−1^ DM). The dominant compound in both distillation products was thymol, with contents ranging from 51.55% to 68.63% (essential oils) and 90.31 to 100% (hydrosols). The essential oils that we analyzed were characterized by a higher polyphenol content than hydrolates. The highest polyphenol content among the essential oils was found in the flower essential oil (415.84 mg GAE·mL^−1^). All the essential oils tested were characterized by a high antioxidant activity (DPPH IC50 from 0.77 (leaf essential oil) to 0.92 μL (flower essential oil)). The essential oils tested also exhibited a broad spectrum of antimicrobial activity against Gram-positive and Gram-negative bacteria and yeasts, while hydrosols showed selective antifungal activity, without significant antibacterial activity.

## 1. Introduction

Oil-bearing plants, known as aromatic herbs and spices, possess valuable properties and are used in culinary, processing, medicinal, pharmaceutical and cosmetic applications. They are found both on herb plantations and in their natural habitats. For years, their properties have been utilized in folk medicine and increasingly in modern medical applications. Oil-bearing plants are a valuable and diverse source of bioactive substances, including, above all, essential oils [1,2]. Essential oils, natural mixtures of volatile substances and hydrophobic compounds, are secondary metabolites synthesized only by certain plant species. They are obtained through hydrodistillation or steam distillation, and the by-product of this process is hydrosols, which are separated from the essential oil at the end of the distillation process. These products are formed during the same process but differ significantly in their chemical composition and biological activity [3]. Essential oils are hydrophobic mixtures mainly containing terpenes, including monoterpenoids, sesquiterpenoids and phenylpropanoids, whilst hydrosols are hydrophilic aqueous solutions containing diluted (up to a maximum of 1 g/L) terpene compounds from the corresponding essential oil. In hydrosols, the proportion of terpene molecules is determined by their hydrophilic properties; therefore, the main components of an essential oil may differ from those in the corresponding hydrosols [4]. Differences in the ratio of terpenes between the essential oil and the hydrosol of *Satureja hellenica* Halácsy have been demonstrated, which may be linked to differences in their biological activity [5]. In recent years, there has been growing interest in natural products derived from aromatic plants, including essential oils and hydrosols. Whilst there are many scientific articles describing the biological properties and potential applications of essential oils, few studies focus on hydrosols, particularly on comparing the two products obtained through the same distillation process [6]. The key components of essential oils have been recognized for their various therapeutic properties; among other things, they have attracted considerable attention for their potential antimicrobial and antioxidant properties [7,8,9]. The growing problem of antibiotic resistance is prompting the food and pharmaceutical industries to seek alternative, natural sources of antioxidants and substances with antimicrobial properties. Suggestions have emerged to limit the use of synthetic preservatives and replace them with natural, aromatic preservatives, such as essential oils and hydrolates, due to their documented biological activity. The advantages of essential oils (e.g., from Lamiaceae plants) over synthetic preservatives include reduced production of toxic by-products and economic viability [10].

One of the largest and most important families of oil-bearing plants is the Lamiaceae family, many members of which are a source of valuable raw material with antioxidant, anti-inflammatory, antimicrobial, antirheumatic, antidepressant, carminative, neuroprotective, sedative, and tonic properties [1,10,11]. A lesser-known but noteworthy member of this family is the bee balm (*Monarda didyma* L.), which is also distinguished by its considerable ornamental value (Figure S1). *M. didyma* is an aromatic perennial with proven health benefits and a wide range of culinary, medicinal and cosmetic uses. The bee balm flower is often used as a medicine and is also added to dishes as a spice [12]. It was traditionally used by the indigenous peoples of North America to treat infections of the digestive and respiratory systems and skin conditions, as it possesses sedative, anti-inflammatory, antimicrobial and antioxidant properties associated with the presence of thymol and carvacrol in its essential oil [12,13]. The above-ground parts of the bee balm contain up to 3.5% essential oil, mainly in the flowers and leaves. This is an oily liquid with a balsamic-sweet scent and a color ranging from pale yellow to reddish-brown [14]. Eight chemotypes of *M. didyma* essential oil have been identified, containing the following dominant components: cymene, γ-terpinene, linalool, thymol methyl ether, thymol, carvacrol, borneol and geraniol [15,16]. *M. didyma*’s essential oil exhibits a broad spectrum of antimicrobial activity, as confirmed by numerous studies [17,18,19]. It has been demonstrated that the antimicrobial activity of the essential oil from *M. didyma* ‘Scorpio’ was higher than that described in the European Pharmacopoeia for common sage and comparable to that of oregano [20].

The aim of our study was to compare the chemical composition and antioxidant, antibacterial and antifungal activity of the essential oil and hydrosol obtained by the same distillation process from *M. didyma* leaves, flowers and aerial parts. Our hypothesis was that essential oils and hydrosols obtained by the same extraction process from different parts of the plant would differ in quantity and quality. It remained unclear whether variations in the chemical composition of the product obtained from the same distillation process would lead to differences in activity, which is what we aimed to clarify.

## 2. Results

### 2.1. The Content, as Well as the Quantitative and Qualitative Composition, of the Essential Oil and Hydrosol Obtained from the Bee Balm, Determined by GC-MS

During the distillation process, hydrophobic (essential oils) and hydrophilic (hydrosols) fractions were obtained from the leaves, flowers and aerial parts (herb) of bee balm (*M. didyma* L.). The highest yield of essential oil (mL·100 g^−1^ DM) was obtained from the flowers, whilst the highest yield of hydrosol was obtained from the leaves (42.46 mL·100 g^−1^ DM) (Table 1).

A total of 47 compounds were identified in the essential oils studied. The dominant compound in all the essential oils tested was thymol, with a content ranging from 51.55% (EO3) to 68.63% (EO2), followed by 6-ethyl-3,4-dimethylphenol (5.27% EO2–19.98% EO3); 3,7,7-trimethyl-1,3,5-cycloheptatriene (3.24% EO2–7.25% EO3); o-cymene (3.69 EO1–5.75% EO3); γ-terpinene (2.86 EO3–4.52% EO1), terpinene isomer (1.71 EO3–4.35% EO2), p-pymene-2-ol methyl ether (0.15 EO1–3.87% EO3), 2-carene (1.29 EO3–2.34% EO1) and α-terpinolene (0.99 EO3–1.63% EO1) (Table 2).

**Figure 1:**
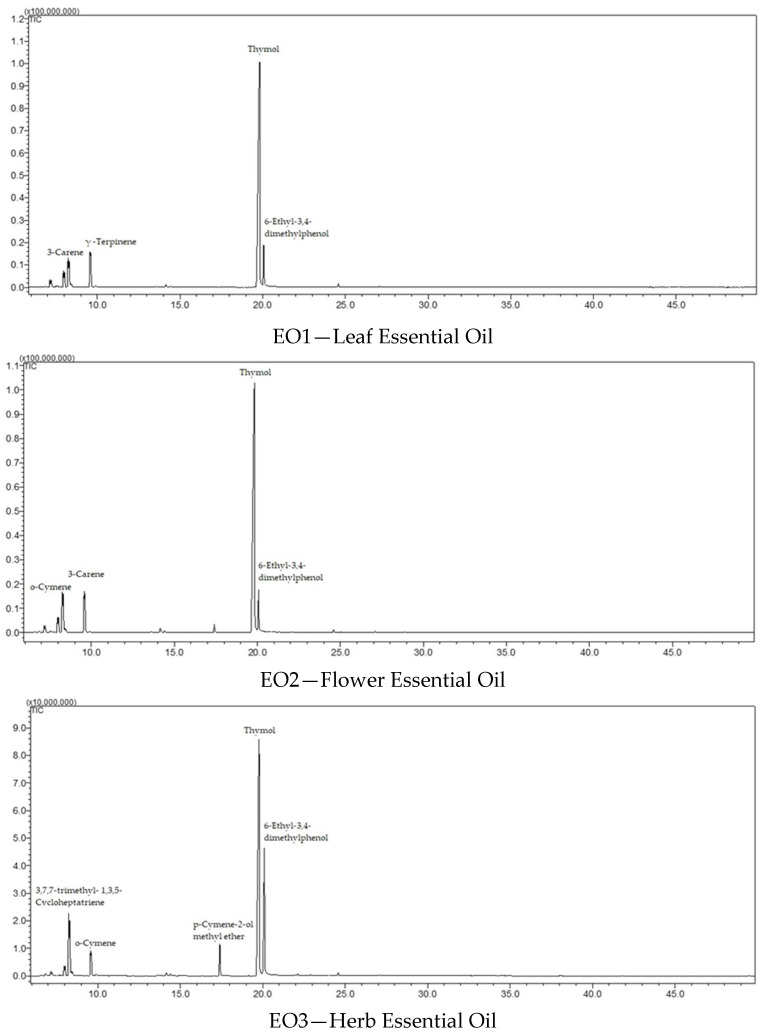
Chromatograms of essential oils obtained from *M. didyma*.

**Figure 2:**
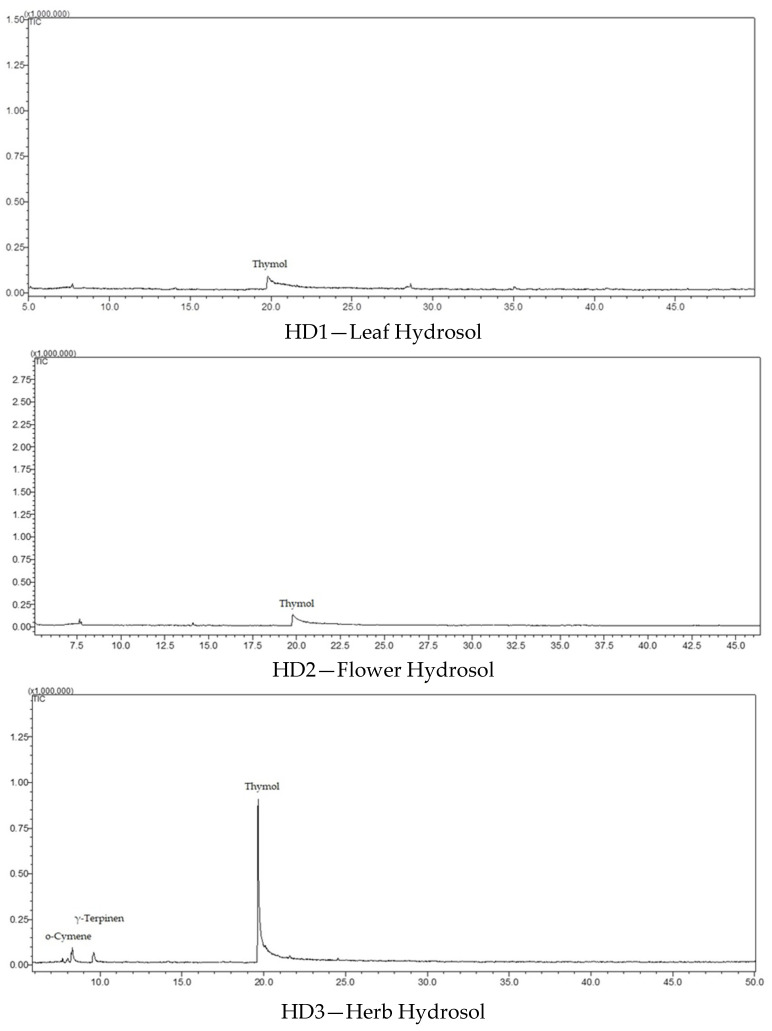
Chromatograms of hydrosols obtained from *M. didyma*.

Thymol was also the main component of the hydrosols (90.31–100%). Analysis of the hydrosols revealed that the hydrosol obtained from the above-ground parts had the most varied chemical composition. The richest fraction turned out to be the hydrosol obtained from the herb, which, in addition to the dominant thymol (90.31%), contained o-cymene (5.36%) and γ-terpinene (4.33%). Figure 1 and Figure 2 show the chromatograms of the essential oils and hydrosols, with the predominant compounds highlighted.

### 2.2. Antioxidant Activity of the Essential Oils and Hydrosols

The complex nature of biological samples necessitates the use of a variety of antioxidant assays. The antioxidant activity (AA) of the essential oils and hydrosols was determined using the DPPH and FRAP methods. The results are expressed as the percentage of DPPH inhibition for the essential oil dilution (1:50) and the undiluted hydrosol (Table 3).

The highest TPC among the essential oils was found in the EO2 flower oil (415.84 mg GAE·mL^−1^), whilst the lowest was in the herb oil (306.82 mg GAE·mL^−1^). The hydrosols showed significantly lower polyphenol contents, ranging from 5.97 mg GAE·mL^−1^ (HD1) to 7.06 mg GAE·mL^−1^ (HD3). All essential oils exhibited a strong ability to scavenge free radicals, ranging from 89.81% (EO2) to 90.63% (EO1). The hydrosols also demonstrated high AA of over 60%, with the hydrosol from the HD3 herb exhibiting the highest activity. To eliminate any errors during the antioxidant assay, including in the preparation of concentrations, calculations and spectrophotometer readings, the range of sample concentrations capable of causing 50% inhibition was determined, along with a regression equation with an R^2^ value close to 1. The IC50 values can be considered reliable. AA is also expressed as the IC50; the results obtained using this method confirm that the essential oils have much stronger antioxidant activity than the hydrosols. All the essential oils were characterized by comparable DPPH IC50s (0.77–0.92 μL). The IC50 value can be defined as the concentration at which the activity of free radicals—specifically, DPPH free radicals—is inhibited by 50%. The lower the IC50 value, the greater the antioxidant activity of the test material. AA measured by the FRAP method is expressed as Trolox equivalents; the strongest FRAP was exhibited by leaf essential oil 43.08 EO1, and the weakest by herb oil 20.55 EO3. In the hydrosol group, the herb hydrosol (HD3) exhibited the strongest FRAP (3.19 mg Tr·g^−1^ DM.), whilst the leaf hydrosol HD1 exhibited the weakest (2.51 mg Tr·g^−1^ DM.).

### 2.3. Antimicrobial Activity of the Essential Oils and Hydrosols

**Figure 3:**
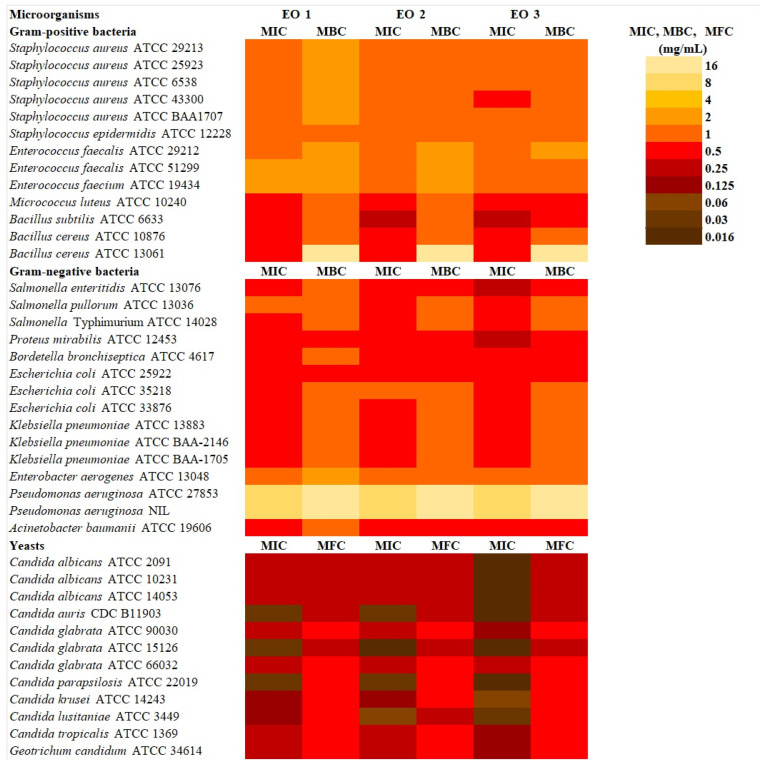
Heatmap of the antimicrobial activity of *Monarda didyma* L. essential oils, expressed as MIC, MBC and MFC values (mg/mL). The colors correspond to the actual concentration values shown on the scale on the right side of the figure. Darker colors indicate lower MIC/MBC/MFC values and, consequently, stronger antimicrobial activity. MBC (minimum bactericidal concentration) values are presented for bacterial strains, while MFC (minimum fungicidal concentration) values are presented for yeasts. EO1—leaf essential oil; EO2—flower essential oil; EO3—herb essential oil.

Essential oils extracted from the leaves (EO1), flowers (EO2) and herb (EO3) of *M. didyma* L. exhibited a broad spectrum of antimicrobial activity against Gram-positive and Gram-negative bacteria and yeasts. The antimicrobial efficacy varied depending on the microorganism tested and the plant part from which the essential oil was extracted (Figure 3).

Among Gram-positive bacteria, the MIC values for all tested essential oils ranged from 0.25 to 1 mg/mL, indicating relatively good to moderate susceptibility in this group of microorganisms. The strongest activity was observed for EO2 and EO3 against *Bacillus subtilis* ATCC 6633, *B. cereus* ATCC 10876, *B. cereus* ATCC 13061, and *Micrococcus luteus* ATCC 10240, for which the lowest MIC values of 0.25–0.5 mg/mL were recorded. In contrast, strains of *Staphylococcus* spp. and *Enterococcus* spp. showed slightly lower susceptibility, with MIC values of 1–2 mg/mL for all the tested essential oils. An exception was the activity of EO3 against *S. aureus* ATCC 43300, with an MIC of 0.5 mg/mL. The MBC values ranged from 0.5 to 16 mg/mL. Analysis of the MBC/MIC ratio showed that the essential oils exhibited mainly bactericidal activity against most Gram-positive strains (MBC/MIC ≤ 4), although bacteriostatic activity (MBC/MIC > 4) was also observed in the case of *B. cereus* ATCC 13061.

Gram-negative bacteria showed lower sensitivity to the tested essential oils compared to Gram-positive microorganisms. The MIC values ranged from 0.25 to 8 mg/mL, while the MBC values ranged from 0.5 to 16 mg/mL. EO3 exhibited the highest antibacterial activity in this group of microorganisms, particularly against *Salmonella enteritidis* ATCC 13076 and *Proteus mirabilis* ATCC 12453, with an MIC of 0.25 mg/mL. In contrast, two strains of *Pseudomonas aeruginosa* exhibited the highest resistance to all the tested essential oils, as evidenced by higher MIC values of 8 mg/mL and MBC values of 16 mg/mL. Analysis of the MBC/MIC ratio demonstrated the bactericidal activity of all the tested essential oils.

Among the tested *M. didyma* essential oils, the strongest antimicrobial activity was observed against yeasts, with MIC and MFC values ranging from 0.016 to 0.25 mg/mL and 0.25 to 0.5 mg/mL, respectively. EO2 and EO3 exhibited the strongest antifungal activity, with MIC values of 0.016 mg/mL against selected *Candida* strains. In particular, EO3 (herb-derived essential oil) showed strong activity (MIC = 0.016 mg/mL) against three *Candida albicans* strains, *C. glabrata* ATCC 15126, and *C. parapsilosis* ATCC 22019, while very good activity (MIC = 0.03–0.06 mg/mL) was observed against *C. auris* CDC B11903, *C. lusitaniae* ATCC 3449, and *C. krusei* ATCC 14243. EO2 demonstrated similarly high efficacy, especially against *C. glabrata* ATCC 15126 (MIC = 0.016 mg/mL), as well as *C. auris* CDC B11903, *C. parapsilosis* ATCC 22019, and *C. lusitaniae* ATCC 3449 (MIC = 0.03–0.06 mg/mL). Notably, *C. auris*, *C. glabrata* and *C. parapsilosis* were the most susceptible strains across all the tested oils, with MIC values consistently ranging from 0.016 to 0.03 mg/mL. Analysis of the MFC/MIC ratios indicated predominantly fungicidal activity at higher MIC values (0.125–0.25 mg/mL; MFC/MIC = 1–4), whereas lower MIC values (0.016–0.06 mg/mL) were generally associated with fungistatic effects (MFC/MIC = 8–16).

Overall, EO2 and EO3 consistently exhibited stronger antimicrobial activity than EO1, particularly against fungal pathogens. These differences appear to be closely related to qualitative and quantitative differences in chemical composition, especially in the content of thymol and associated monoterpenes.

The susceptibility of the reference strains to standard antimicrobial agents was confirmed by MIC values of 1 µg/mL for fluconazole, 1 µg/mL for vancomycin and 0.015 µg/mL for ciprofloxacin against *C. albicans* ATCC 10231, *S. aureus* ATCC 29213 and *E. coli* ATCC 25922, respectively.

The study also evaluated the antimicrobial activity of three hydrosols obtained from different parts of *M. didyma* L.—the leaves (HD1), flowers (HD2) and herb (HD3)—against selected yeast strains using the well diffusion method. The antimicrobial activity of these hydrosols is expressed as the diameter of the inhibition zones (mm) (Table 4). The results indicate a clear variation in the antifungal activity of the tested hydrosols depending on the plant part used to obtain them. The highest activity was exhibited by the herb hydrosol (HD3), which inhibited the growth of most of the tested yeast strains. The zones of growth inhibition for HD3 ranged from 10 to 11 mm and were observed for the strains *C. albicans* ATCC 2091, ATCC 10231, and ATCC 14053; *C. auris* CDC B11903; *C. glabrata* ATCC 15126; *C. parapsilosis* ATCC 22019; and *C. lusitaniae* ATCC 3449. The flower hydrosol (HD2) exhibited limited antifungal activity, observed only against *C. glabrata* ATCC 15126, for which the diameter of the growth inhibition zone was 11 mm. In contrast, the leaf hydrosol (HD1) showed no biological activity against *Candida* spp. It is worth noting that some of the tested yeast strains, including *C. glabrata* ATCC 90030 and ATCC 66032, *C. krusei* ATCC 14243, *C. tropicalis* ATCC 1369, and *Geotrichum candidum* ATCC 34614, showed complete resistance to all of the analyzed hydrosols.

At the same time, the activity against Gram-positive and Gram-negative bacteria was evaluated; however, none of the tested hydrosols exhibited inhibitory activity against the bacterial strains analyzed. These results suggest that hydrosols derived from *M. didyma* L. exhibit selective antifungal activity, while showing no significant antibacterial activity.

## 3. Discussion

*M. didyma*, as a medicinal aromatic plant, is a valuable source of essential oil. However, the chemical composition of bee balm essential oil is not constant and depends on various factors. Wróblewska and others [20] identified the following compounds as the main constituents in the essential oil of *M. didyma*: linalool, p-cymene, thymol and thymol methyl ether. The main constituents of the *M. didyma* essential oil as determined by Adebayo et al. [21] were thymol (41.17%), γ-terpinene (15.88%), carvacrol (15.20%) and para-myrcene (12.58%), and this oil proved to be the most effective against *Botrytis cinerea*, compared with essential oils obtained from other *Monarda* plants. Ontogenetic factors are among the most important that modify the chemical composition of *M. didyma* essential oil. Essential oil obtained from different parts of the plant (leaf, flower) may differ in quantity and quality. Gontar et al. [14], meanwhile, report essential oil contents in the leaves and inflorescences of *M. didyma* collected during the flowering period of 2.39% and 3.64%, respectively, with a predominance of p-cymene (17.11–35.56%) and carvacrol (5.39–28.19%). After analyzing the leaves and flowers, the authors found that the inflorescences contained the highest levels of essential oil, p-cymene and carvacrol. Our results are consistent, though not in terms of the oil’s chemical composition. However, we confirmed that the flower oil had the highest thymol content compared to the others. The *M. didyma* essential oil and hydrosol we examined exhibited the thymol chemotype, corresponding to that described by Italian researchers [22], which was also confirmed for *M. citriodora* Cerv. ex Lag [23], and demonstrated marked biological activity.

The biological activity of *M. didyma* essential oil is attributed to the presence of thymol [24,25] or carvacrol [15]. Essential oils and hydrosols obtained by distillation from the same plant do not always have the correct chemical composition [26,27]. The differences in the chemical composition of the essential oils and hydrosols that we have identified are due to the compounds’ lower solubility in water. In the products we obtained, the thymol content was higher in the hydrosols than in the essential oils. Similarly, Hay et al. [28] showed a higher thymol content (98.1%) in thyme hydrosol than in thyme essential oil (36.1%). Thymol has poor solubility in water (about 1 g·L^−1^ at 20 °C) but dissolves readily in alcohols and organic solvents [29]. The relatively high level of this compound in hydrosols can be attributed to its stronger affinity for water compared with other constituents of the essential oil. Pardavella et al. [5] demonstrated that oxygenated monoterpenes are extracted more efficiently into the hydrosol from *Saturej hellenica* than monoterpene hydrocarbons, which is explained by their hydrophobic nature.

Antioxidants are substances that, even at low concentrations, prevent oxidation or markedly slow down the oxidation of a substrate. The primary sources of antioxidants are plant-derived products and raw materials [30]. Currently, the most intensive research directions for hydrosols relate to chemical composition, antioxidant activity and antimicrobial activity [31]. The different chemical compositions of essential oils and hydrosols make them distinct products with specific purposes. Essential oils generally exhibit stronger biological activity than hydrosols [4,32]. However, the activity of these products depends on their chemical composition. Hydrosols produced from *Hyssopus officinalis*, *Marrubium vulgare* and *Artemisia herba-alba* demonstrate good antioxidant activity. However, the hydrosol produced by the hydrodistillation of the resinous part of *Aquilaria* sp. wood exhibited very low antioxidant activity compared to quercetin [4]. Hydrocarbonated terpenes (limonene, β-caryophyllene, pinenes) are nonpolar and do not bind to water molecules; hence, they are found almost exclusively in essential oils and rarely in hydrosols. Hydrosols, on the other hand, are rich in oxygenated compounds that are more soluble in or have a stronger affinity for water [32]. Hydrosols rich in phenolic compounds, such as thymol and carvacrol, have stronger antioxidant activity than others [33]. Thymol and carvacrol, as phenolic monoterpenoids, reduce DPPH radicals, transforming them into a reduced (non-radical) form [34]. The assessment of this activity depends, among other things, on the chosen method. A range of analytical methods has been used to assess the antioxidant potential of plant extracts. Two categories of methods are used to determine antioxidant activity (AA): the reduction of metal ions to ions with a lower oxidation state by the antioxidant under investigation (FRAP), and the scavenging of free, stable radicals (DPPH). The FRAP method covers the majority of antioxidant components in the sample, whilst the DPPH method covers only some of the most reactive ones. The results of the DPPH assay are commonly reported as the IC50 value, which represents the concentration of antioxidant needed to scavenge 50% of the free radicals [35]. A comparative evaluation using different antioxidant assessment methods indicates that they are not all strongly correlated; therefore, antioxidant capacity should be measured using multiple approaches [36,37]. In addition, the high correlation between the FRAP, TEAC and DPPH assays (r = 0.844–0.907) highlights their complementary nature in capturing different aspects of antioxidant activity [38]. Our findings support this argument. The DPPH values, expressed as percentages of inhibition, are preliminary; it was only after determining the IC50 that the potential of the essential oils and hydrosols could be properly assessed. This was subsequently confirmed by FRAP. The DPPH test is sensitive to samples containing phenolic compounds and derivatives [32]. Our results indicate the antioxidant potential of both products obtained from the distillation of the leaves, flowers and herbs of *M. didyma*. When analyzing the antioxidant activity of the tested hydrosols, it should be noted that the hydrosols obtained from the above-ground parts (herb), containing, in addition to thymol, o-cymene and γ-terpinene, showed the highest activity in the DPPH and FRAP tests. Monoterpenes such as γ-terpinene exhibit significant pharmacological effects. This compound, one of the key terpenes responsible for the antioxidant activity of oils, has been shown to reduce the peroxidation of methyl linoleate and egg yolk phospholipids, as well as the peroxidation of low-density lipoproteins [39,40].

Essential oils obtained from different parts of *M. didyma* L. demonstrated strong antimicrobial activity, particularly against yeasts and Gram-positive bacteria. The observed effects depended on both the tested microorganisms and the chemical composition of the oils. Particularly low MIC values against *Candida* spp., including multidrug-resistant *Candida auris*, indicate high antifungal potential and suggest possible applicability against resistant fungal infections [16,41].

GC-MS analysis identified thymol as the dominant constituent in all tested oils (51.55–68.63%), indicating its major contribution to the antimicrobial activity. Thymol is known to disrupt microbial membrane integrity, increase permeability, and induce leakage of intracellular components [41]. The stronger antimicrobial activity observed for EO2 and EO3 may be related to their high thymol content and may be partly explained by possible interactions between thymol and other secondary constituents, including γ-terpinene and p-cymene [41]. However, further studies using isolated compounds and combination tests are needed to confirm potential synergistic effects. *Coridothymus capitatus* (L.) Rchb. hydrosol, whose dominant component was carvacrol (thymol isomer), demonstrated good antimicrobial activity against bacteria and yeasts. Synergistic activity with itraconazole against *Candida krusei* and additive activity with tetracycline against methicillin-resistant Staphylococcus aureus strains were demonstrated. The hydrolate altered the permeability of bacterial and yeast cell membranes and disrupted mitochondrial function [42].

The lower susceptibility of Gram-negative bacteria compared with Gram-positive strains is likely associated with the structural properties of their outer membrane, which limits penetration of hydrophobic compounds. This may explain the weaker activity observed against *Pseudomonas aeruginosa* and *Acinetobacter baumannii*.

There are suggestions regarding the mechanism of action of essential oils. The results of Chen et al. [17] suggest that *M. didyma* essential oil may exert an inhibitory effect on *Carbapenem-resistant Klebsiella pneumoniae* strains, and the potential mechanisms of action include inhibition of biofilm formation, damage to cell membrane structure, and inhibition of energy metabolism. The anti-biofilm action is particularly important here, suggesting the exceptional effectiveness of the essential oil in the fight against pathogenic microorganisms.

The obtained results suggest potential applications of *M. didyma* essential oils in pharmaceutical, cosmetic, and food-related products, particularly as natural antifungal and antimicrobial agents.

The present findings indicate that hydrosols derived from *M. didyma* L. exhibit selective antifungal activity against *Candida* spp., whereas no activity was observed against Gram-positive or Gram-negative bacteria. Among the tested samples, the herb hydrosol (HD3) showed the strongest antifungal effect, which may be associated with the higher abundance of biologically active volatile metabolites in the aerial parts of the plant.

Recent studies indicate that hydrosols should not be regarded solely as by-products of essential oil distillation, but also as valuable sources of bioactive compounds with potential biological applications [43]. The antifungal activity observed for HD3 is likely related to the presence of thymol (90.31%) together with o-cymene (5.36%) and γ-terpinene (4.33%), compounds characteristic of the Lamiaceae family and known for their membrane-disrupting effects against *Candida* spp. In contrast, HD1 and HD2, despite containing thymol as the dominant constituent, did not exhibit comparable activity, suggesting that synergistic interactions between major and minor components play an important role in antifungal efficacy. Similar synergistic effects among monoterpenes have been previously described for essential oils and hydrosols rich in phenolic compounds [6,44].

Of particular importance is the activity of HD3 against *Candida auris*, an emerging multidrug-resistant pathogen associated with biofilm formation and limited therapeutic options. These findings support the growing interest in plant-derived products as potential alternative antifungal agents [45]. The selective antifungal activity and potentially milder biological profile of hydrosols compared with essential oils may also support their application in cosmetic and dermocosmetic formulations, particularly those intended for sensitive or microbiome-imbalanced skin.

## 4. Materials and Methods

### 4.1. Plant Material

The test material consisted of essential oils (EOs) and hydrosols (HDs) obtained from the aerial parts of bee balm (*Monarda didyma* L.) plants. The plants were grown at the Felin Experimental Farm of the University of Life Sciences in Lublin in the 2025 season, in a sunny location, naturally sheltered from the wind by a row of trees and shrubs. The seeds came from the seed company W. Legutko Hobby Sp. z. o.o., (Rawicz, Poland). The crop was established from seedlings produced in an unheated greenhouse, and the plants were planted out in the first ten days of June 2025. No chemical plant protection products were used during the growing season; no diseases or pests were observed, and systematic manual weeding was carried out. During the summer months of July and August, the plant material was harvested, comprising leaves, flowers and herbs. The collection of raw materials followed the general principles of herb harvesting: leaves were collected before flowering, and flowers and herbs (above-ground parts) in full bloom. The harvested fresh plant material was weighed and sent for drying in a thermal dryer at a temperature of 35 °C. Drying was carried out at a constant temperature for 2 days; then, the plant material was sent for the distillation of essential oils (Table 5).

### 4.2. Reagents and Reference Strains

Reference strains of bacteria and fungi from the American Type Culture Collection (ATCC, LGC Standards, Teddington, UK), the Centers for Disease Control and Prevention (CDC, Atlanta, GA, USA), and the National Institute of Medicines (NIL, Warsaw, Poland) were used in the study. A total of 28 bacterial reference strains were included: thirteen Gram-positive bacteria (*Staphylococcus aureus* ATCC 25923, 29213, and 6538—methicillin-susceptible strains; *S. aureus* ATCC 43,300 and BAA-1707—methicillin-resistant strains; *S. epidermidis* ATCC 12228; *Enterococcus faecalis* ATCC 29212 and 51299; *E. faecium* ATCC 19434; *Micrococcus luteus* ATCC 10240; *Bacillus subtilis* ATCC 6633; *B. cereus* ATCC 10,876 and 13061) and fifteen Gram-negative bacteria (*Salmonella enteritidis* ATCC 13076; *S. pullorum* ATCC 13036; *S.* Typhimurium ATCC 14028; *Proteus mirabilis* ATCC 12453; *Bordetella bronchiseptica* ATCC 4617; *Escherichia coli* ATCC 25922, 35,218 and 33876; *Klebsiella pneumoniae* ATCC 13883, BAA-2146 and BAA-1705; *Enterobacter aerogenes* ATCC 13048; *Pseudomonas aeruginosa* ATCC 27853; and the *P. aeruginosa* NIL clinical reference strain, *Acinetobacter baumannii* ATCC 19606). Twelve reference yeast strains were used in the study: *Candida albicans* ATCC 2091, 10231 and 14053; *C. auris* CDC B11903; *C. glabrata* ATCC 90030, 15126 and 66032; *C. parapsilosis* ATCC 22019; *C. krusei* ATCC 14243; *C. lusitaniae* ATCC 3449; *C. tropicalis* ATCC 1369; and *Geotrichum candidum* ATCC 34614. Ciprofloxacin, vancomycin and fluconazole (Sigma-Aldrich, St. Louis, MO, USA; Becton, Dickinson and Company, Franklin Lakes, NJ, USA; Oxoid™ Thermo Fisher Scientific, Altrincham, UK; and BioMaxima, Lublin, Poland, respectively) were used as positive controls in both the broth serial microdilution and well-diffusion method.

### 4.3. The Distillation of Essential Oils and Hydrosols

The essential oils were extracted by steam distillation using a Clevenger apparatus. For the distillation, a 20 g sample of plant material, including leaves, flowers and herbs, was taken and placed in a round-bottomed flask, to which 450 mL of distilled water was added. The mixture was brought to the boil and the distillation rate was adjusted. Distillation was carried out for 3 h. After this time, the heating jacket was switched off and left for 15 min to allow the level of essential oil in the receiver to stabilize. The distillation process was carried out in three replicates. After the elapsed time, the hydrosol and essential oil were brought down to the microscale, the volume was read in mL, and the samples were collected into dark glass vials. The hydrosol and essential oil samples were stored in a refrigerator at 4 °C until the chemical profile was assessed.

### 4.4. GC-MS Analysis of Essential Oils and Hydrosols

Prior to GC–MS analysis, 10 μL of essential oil or 30 μL of hydrosol was diluted with methanol to a final volume of 1.0 mL. Subsequently, 1 μL of the prepared solution was injected into the GC–MS system. Chromatographic analysis of essential oils and hydrosols was performed using a GC/MS QP2010 (Shimadzu, Kyoto, Japan) equipped with a ZB-5MSi fused-silica capillary column (30 m × 0.25 mm i.d., 0.25 μm film thickness; Phenomenex; 411 Madrid Ave, Torrance, CA, USA). Helium (5.0 grade) was used as the carrier gas at a flow rate of 1 mL min^−1^. The injector temperature was maintained at 310 °C, and analyses were performed in split mode (purge time 0.7 min). The oven temperature program was as follows: 50 °C for 2 min, followed by a linear increase at 5 °C min^−1^ to 310 °C. The mass spectrometer operated in electron ionization mode (70 eV). The ion source temperature was 220 °C, and spectra were acquired over the mass range *m*/*z* 35–450. Compounds were identified by comparing the mass spectra and retention indices with data available in the NIST/EPA/NIH Mass Spectral Library [46] and the published literature. The relative composition was estimated using the peak area normalization method and is expressed as the percentage peak area of the total ion chromatogram.

### 4.5. Determination of Total Polyphenol Content (TPC)

The Folin–Ciocâlteu method, with minor modifications [47,48], was used to determine the TPC in essential oils and hydrosols. Hydrosols were analyzed undiluted, whilst essential oils were diluted in methanol (POCH, Gliwice, Poland) at a ratio of 1:400. For the determination, 0.1 mL of the sample was taken and mixed with 6 mL of distilled water and 0.5 mL of Folin–Ciocâlteu reagent (POCH, Gliwice, Poland); the mixture was stirred and left to stand for 3 min. After this time, 1.5 mL of a saturated sodium carbonate solution (Chempur, Piekary Śląskie, Poland) and 1.9 mL of distilled water were added, and the mixture was placed in a water bath (40 °C) for 30 min. The absorbance was then measured at a wavelength of 765 nm, using a mixture of reagents without the extract as a reference. The measurements were performed using a Hitachi U-2900 UV-Vis spectrophotometer (PerkinElmer, Waltham, MA, USA). The results were calculated based on a standard curve for gallic acid (GA) and are expressed as mg of polyphenolic compounds per mL of product, calculated as GA.

### 4.6. Assessment of Antioxidant Activity (AA) Using the DPPH Method

The AA of essential oils and hydrosols was assessed using the DPPH method, as described by Yen and Chen [49]. This method involves the colorimetric measurement of the degree of reduction of DPPH free radicals (2,2-diphenyl-1-picrylhydrazyl, Merck, Poznań, Poland). To prepare the DPPH (2,2-diphenyl-1-picrylhydrazyl) solution, 0.012 g of DPPH was weighed and quantitatively transferred to a 100 mL volumetric flask, filled to the mark with methanol (100%), and then dissolved in an ultrasonic bath for 15 min. A 1 mL volume of DPPH solution was used for analyses. The absorbance was measured using a Hitachi U-2900 UV–Vis spectrophotometer (Hitachi High-Technologies Company; Tokyo, Japan), at a wavelength of 517 nm, using a reference solution (methanol) (POCH, Gliwice, Poland). To determine the DPPH activity, three dilutions of the essential oil (1:50; 1:2500; 1:25,000) and hydrosols (1:0; 1:1; 1:3) in methanol were prepared and used for the test. Measurements were taken for the three dilutions; all analyses were carried out in triplicate. Decreasing absorbance levels indicated increased antioxidant activity of the tested essential oils and hydrosols. The assessed antioxidant activity is expressed as the percentage of free radical inhibition by the tested essential oils and hydrosols (%) and the concentration of essential oils and hydrosols that destroyed 50% of the free radicals in the tested samples (IC50). To determine the IC50, a standard curve was plotted, based on which the amounts of essential oils and hydrosols were determined.

### 4.7. Determination of AA Using the FRAP Method

The AA-FRAP values were determined for the essential oils and hydrosols in accordance with the method described by Thaipong et al. [50] and Mulugeta et al. [51], with modifications. To analyze antioxidant activity, 100 μL of appropriately diluted essential oil (1:25,000) and hydrosol (1:3) were taken to determine AA using the FRAP method, as described by Gruszecki et al. [52], with some modifications. AA was assessed by measuring the absorbance at a wavelength of 593 nm and using a blank sample containing a mixture of reagents without the test samples, using a Hitachi U-2900 UV-Vis spectrophotometer (Hitachi High-Technologies Company; Tokyo, Japan). The results were read from a calibration curve for a standard Trolox solution. The antioxidant capacity of the samples is expressed as Trolox equivalents (mg Trolox g^−1^ dry weight).

### 4.8. Assessment of the Microbiological Activity

#### 4.8.1. The Serial Microdilution Method

The antimicrobial activity of *Monarda didyma* L. extracts was evaluated using the broth microdilution method according to the guidelines of the European Committee on Antimicrobial Susceptibility Testing (EUCAST) [53]. The minimum inhibitory concentration (MIC) was determined against 40 reference bacterial and yeast strains obtained from the American Type Culture Collection (ATCC), the Center for Disease Control and Prevention (CDC), and the National Institute of Medicines (NIL, Warsaw, Poland). Prior to testing, bacterial strains were subcultured on Mueller–Hinton agar (MHA) and incubated at 35 ± 1 °C for 18 ± 2 h. Yeast strains were cultured on Sabouraud dextrose agar (SDA) and incubated at 35 °C for 24 h. Microbial colonies were suspended in sterile saline solution to achieve an inoculum density equivalent to a 0.5 McFarland standard, corresponding to approximately 1.5 × 10^8^ CFU/mL for bacteria and 5 × 10^6^ CFU/mL for yeasts. The inoculum suspensions were further diluted in broth medium to obtain a final inoculum of approximately 5 × 10^5^ CFU/mL in each well.

The tested extracts were dissolved in dimethyl sulfoxide (DMSO) to obtain stock solutions at a concentration of 1000 mg/mL. The final concentration of DMSO in Mueller–Hinton broth (MHB) or RPMI 1640 broth buffered with MOPS did not exceed 3.2% at the highest tested extract concentration (16 mg/mL) and decreased proportionally with each subsequent twofold dilution. Serial twofold dilutions of the extracts were prepared in sterile 96-well polystyrene microplates using MHB for bacterial strains and RPMI 1640 broth buffered with MOPS for yeasts. The final extract concentrations ranged from 16 to 0.016 mg/mL. The following controls were included in each experiment: medium sterility control, microbial growth control, extract sterility control, and DMSO control. DMSO controls confirmed the absence of antimicrobial activity at the tested concentrations. MIC values were determined visually and confirmed spectrophotometrically using an ELx800 microplate reader (BioTek Instruments Inc., Winooski, VT, USA) at 600 nm. The minimum bactericidal concentration (MBC) and minimum fungicidal concentration (MFC) were additionally determined.

To determine the MBC and MFC, 5 μL of the contents of the wells corresponding to the MIC value, as well as from two concentrations above and two below the MIC, were aseptically collected and inoculated onto MHA (bacteria) or SDA (yeast) media. The inoculated plates were incubated at 35 ± 1 °C for 18 ± 2 h for bacteria and at 35 °C for 24 h for yeasts. The MBC and MFC values were defined as the lowest concentrations resulting in a 99.9% reduction in viable microorganisms compared with the initial inoculum. All experiments were performed in triplicate. To determine the MIC, MBC and MFC, the modal value (the most frequently occurring result) obtained from three independent experiments is reported. Vancomycin, ciprofloxacin and fluconazole were used as reference antimicrobial agents against Gram-positive bacteria, Gram-negative bacteria, and yeasts, respectively. The tested concentration ranges were 0.06–16 µg/mL for vancomycin and fluconazole and 0.015–16 µg/mL for ciprofloxacin.

#### 4.8.2. Well Diffusion Method

The well diffusion test was performed on Mueller–Hinton agar (MHA; for bacteria) and Mueller–Hinton agar with 2% glucose (MHA + 2%; for fungi) in accordance with EUCAST recommendations. The procedure was based on the disk diffusion method used to determine the susceptibility of microorganisms to antibiotics. Suspensions of the same microorganisms as in the serial microdilution method in broth were used for the tests. The inoculum of each microorganism was applied evenly in three directions onto the above agar plates, all of which were 4 mm thick, using a sterile swab dipped in inoculum with a turbidity of 0.5 on the McFarland scale (1.5 × 10^8^ CFU/mL for bacteria, 5 × 10^6^ CFU/mL for yeast). Next, using a cork borer, four 8 mm diameter wells were cut into the agar medium at equal intervals to ensure that the growth inhibition zones did not overlap. Then, 100 µL of hydrosols were added to 3 of them—from leaves (HD1), flowers (HD2) and herbs (HD3)—while the fourth well served as a control to which sterile distilled water was added. The following antibiotics were used as positive controls: ciprofloxacin (5 µg/mL; Becton, Dickinson and Company, USA) for Gram-negative bacteria, vancomycin (30 µg/mL; Oxoid™ Thermo Fisher Scientific, UK) for Gram-positive bacteria, and fluconazole (25 µg/mL; BioMaxima, Poland) for yeast. The plates were left at room temperature until the hydrosols had absorbed into the medium and incubated at 37 °C for 24 h. After incubation, the diameters of the microbial growth inhibition zones (including the well diameters) were measured, and the results are reported in millimeters. Each test was performed three times to ensure the reproducibility of the results, and the mean values were calculated. All procedures were performed under aseptic conditions to prevent contamination. All materials and instruments were sterilized prior to use, and culture plates were stored and used under aseptic conditions.

### 4.9. Statistical Analysis

The results obtained are presented as means and were subjected to statistical analysis using ANOVA. The means were compared using Tukey’s HSD test at a significance level of α = 0.05. Statistical analyses were performed using Statistica 13.3 PL (StatSof Inc., Tulsa, OK, USA). A statistical report containing measures of variability (mean ± SD/SE) is provided in Table S1.

## 5. Conclusions

Essential oils and hydrosols obtained from the leaves, flowers and aerial parts of bee balm (*M. didyma* L.) differed in chemical composition, with thymol predominating in both products. The essential oil from the flowers showed the strongest activity in the TPC method, while the essential oil from the leaves showed the strongest activity in the FRAP method, compared to the others. The hydrosol from the aerial parts of bee balm, containing, in addition to thymol, o-cymene and γ-terpinene, was characterized by stronger activity in the DPPH and DPPH IC50 tests than the other hydrosols.

*M. didyma* hydrosol, as a by-product of the essential oil distillation process, was characterized by a high thymol content and may have potential practical applications. Further research should be directed towards the analysis of the chemical composition and biological activity of essential oils and hydrosols from various organs of other aromatic plants, the possibilities for the effective use of both products, and improving the distillation process using supporting methods.

The distillation products of *M. didyma* leaves, flowers and herbs that we analyzed differed in their chemical composition and activity towards the tested bacteria and fungi. The pronounced antibacterial and antifungal activity of bee balm essential oils, with an almost complete lack of activity of hydrosols (especially against bacteria), indicates the possibility of further research on the assessment of the antimicrobial activity of mixtures of oils and hydrosols. According to research on thyme, the combination of essential oils and hydrosols, compared to single extracts, causes a four-fold reduction in MBC against *Escherichia coli* [28].

## Figures and Tables

**Table 1:** Type of plant material distilled and results of the distillation process.

Distillation Product	Plant Material	Sample Name	Content (mL·100 g^−1^ DM)
Essential Oil	Leaf	EO1	2.46 b
	Flower	EO2	3.71 a
	Herb	EO3	2.84 b
Hydrosol	Leaf	HD1	42.46 C
	Flower	HD2	41.44 D
	Herb	HD3	41.84 D

The results are presented as means. Averages marked with the same letter do not differ significantly from each other at *p* < 0.05.

**Table 2:** Chemical composition of essential oils and hydrosols.

Compound	RI	RT (min)	Content (%)					
			EO1	EO2	EO3	HD1	HD2	HD3
α-Pinene	939	5.79	-	0.05	-	-	-	-
2-Octen-1-ol	968	6.86	0.17	0.33	0.28	-	-	-
β-Myrcene	987	7.14	0.81	0.64	0.35	-	-	-
β-Pinene	979	7.22	1.08	0.99	0.49	-	-	-
Thujene isomer	987	7.51	0.13	0.12	-	-	-	-
α-Phellandrene	1004	7.59	0.17	0.13	-	-	-	-
β-Ocimene	1001	7.71	0.04	-	-	-	-	-
α-Ocimene	1010	7.78	0.08	-	-	-	-	-
α-Terpinolen	1023	7.95	1.63	1.57	0.99	-	-	-
2-Carene	1026	8.02	2.34	2.15	1.29	-	-	-
3,7,7-trimethyl-1,3,5-Cycloheptatriene	1027	8.23	3.24	3.7	7.25	-	-	-
o-Cymene	1034	8.29	3.69	5.54	5.75	-	-	5.36
Limonene	1038	8.45	0.48	0.50	0.59	-	-	-
β-cis-Ocimene	1042	9.31	0.03	0.03	-	-	-	-
γ-Terpinene	1060	9.56	4.52	4.10	2.86	-	-	4.33
Terpinene isomer	1061	9.60	4.14	4.35	1.71	-	-	-
trans-4-Thujanol	1089	9.91	0.31	0.24	0.28	-	-	-
Terpinolene	1098	10.78	0.06	0.07	0.05	-	-	-
4-Thujanol	1118	11.16	0.06	0.05	0.06	-	-	-
Linalool	1120	11.40	0.04	0.08	0.05	-	-	-
Carveol	1124	13.12	0.01	-	-	-	-	-
Pinocamphone	1143	13.61	0.16	0.22	0.20	-	-	-
endo-Borneol	1158	13.86	0.05	0.06	0.06	-	-	-
Isocamphopinone	1170	14.15	0.54	0.76	0.54	-	-	-
cis-Sabinene hydrate	1188	14.41	0.20	0.31	0.34	-	-	-
α-Terpineol	1220	15.09	0.03	0.04	0.05	-	-	-
p-Cymene-2-ol methyl ether	1248	17.42	0.15	1.09	3.87	-	-	-
Thymol	1292	19.82	68.63	66.5	51.55	100.00	100.00	90.31
6-Ethyl-3,4-dimethylphenol	1310	20.07	6.03	5.27	19.98	-	-	-
3-tert-Butylated hydroxyanisole	1348	22.01	-	0.06	0.10	-	-	-
α-Cubebene	1376	22.16	0.03	0.04	0.23	-	-	-
α-Copaene	1388	22.87	0.03	0.03	0.15	-	-	-
β-Bourbonene	1418	23.20	0.02	0.04	0.03	-	-	-
Guaia-3,9-diene	1432	24.22	-	-	0.01	-	-	-
Caryophyllene	1456	24.58	0.50	0.41	0.40	-	-	-
β-Copaene	1483	25.01	0.06	0.05	0.04	-	-	-
Isogermacrene D	1492	25.62	0.01	0.01	-	-	-	-
cis-α-Bisabolene	1494	25.96	0.03	0.03	0.03	-	-	-
4-Methylene-1-methyl-2-(2-methyl-1-propen-1-yl) -1-vinyl-cycloheptane	1498	26.24	0.02	0.02	0.02	-	-	-
γ-Amorphene	1499	26.99	0.06	0.05	0.04	-	-	-
Germacrene D	1506	27.09	0.17	0.15	0.10	-	-	-
Guaia-1(10),11-diene	1508	27.71	0.06	0.06	0.05	-	-	-
α-Muurolene	1510	27.97	-	0.02	-	-	-	-
Tau-Cadinol acetate	1539	28.44	0.05	0.04	0.03	-	-	-
Isoledene	1546	28.87	0.10	0.08	0.06	-	-	-
Elemol	1552	29.88	0.04	0.02	0.07	-	-	-
Humulen-(v1)	1685	30.95	-	-	0.05	-		
Total			100.00	100.00	100.00	100.00	100.00	100.00

RI—Retention Index; RT—Retention Time; EO1—Leaf Essential Oil; EO2—Flower Essential Oil; EO3—Herb Essential Oil; HD1—Leaf Hydrosol; HD2—Flower Hydrosol; HD3—Herb Hydrosol.

**Table 3:** The antioxidant activity of distillation products from *M. didyma*.

Distillation Product	Plant Material	Sample Name	TPC mg GAE·mL^−1^	DPPH %	DPPH IC50 (μL)	FRAP mg Tr·g^−1^ DM
Essential Oil	Leaf	EO1	388.88 b	90.63 a	0.77 a	43.08 a
	Flower	EO2	415.84 a	89.81 a	0.84 a	39.34 b
	Herb	EO3	306.82 c	90.34 a	0.92 a	20.55 c
Hydrosol	Leaf	HD1	5.97 d	62.76 c	408.19 c	2.51 d
	Flower	HD2	5.99 d	61.71 c	426.02 c	2.94 d
	Herb	HD3	7.06 d	72.31 b	361.59 b	3.19 d

TPC—Total phenolic content. The results are presented as means. Letters in columns indicate statistically significant differences in Tukey’s test at a significance level of *p* < 0.05. Means marked with the same letter do not differ significantly.

**Table 4:** Inhibition zone diameters of *M. didyma* L. hydrosols against *Candida* spp. determined using the agar well diffusion method (HD1—Leaf Hydrosol; HD2—Flower Hydrosol; HD3—Herb Hydrosol).

Yeasts	Inhibition Zones [mm]		
	HD1	HD2	HD3
*Candida albicans* ATCC 2091	0	0	11
*Candida albicans* ATCC 10231	0	0	11
*Candida albicans* ATCC 14053	0	0	11
*Candida auris* CDC B11903	0	0	10
*Candida glabrata* ATCC 90030	0	0	0
*Candida glabrata* ATCC 15126	0	11	11
*Candida glabrata* ATCC 66032	0	0	0
*Candida parapsilosis* ATCC 22019	0	0	11
*Candida krusei* ATCC 14243	0	0	0
*Candida lusitaniae* ATCC 3449	0	0	10
*Candida tropicalis* ATCC 1369	0	0	0
*Geotrichum candidum* ATCC 34614	0	0	0

**Table 5:** Characteristics of the research material obtained from the *M. didyma* plants.

Raw Material	Harvest Period	Fresh Matter	Air-Dry Matter
		kg	
Leaves (*folium*)	Before flowering	3.27	1.13
Flowers (*flos*)	Beginning of full bloom	1.86	0.46
Above-ground parts—herb (*herba*)	Beginning of full bloom	2.29	0.62

Biological samples (plant material) were collected from 20 plants; after drying the material, three biological samples were randomly selected for chemical analysis. All chemical analyses were performed in three replicates (technical samples).

## Data Availability

The raw data supporting the conclusions of this article will be made available by the authors on request.

## Supplementary Materials

The following supporting information can be downloaded at https://www.mdpi.com/article/10.3390/molecules31132252/s1, Figure S1: M. didyma plants used as the research material. The photograph shows plants in full flowering, when the flowers and above-ground parts were collected. Table S1: The statistical report containing measures of variability (mean ± SD/SE).
