# Supplementary material for: Chemical Composition, and Antioxidant and Antimicrobial Properties of Monarda didyma L.’s Essential Oils and Hydrosols
Source: Molecules. 2026 Jun 26;31(13):2252. doi: 10.3390/molecules31132252 (PMC13362633; doi:10.3390/molecules31132252)
Supplement: Supplementary file 1 [file molecules-31-02252-s001.zip › Figure S1.pdf]

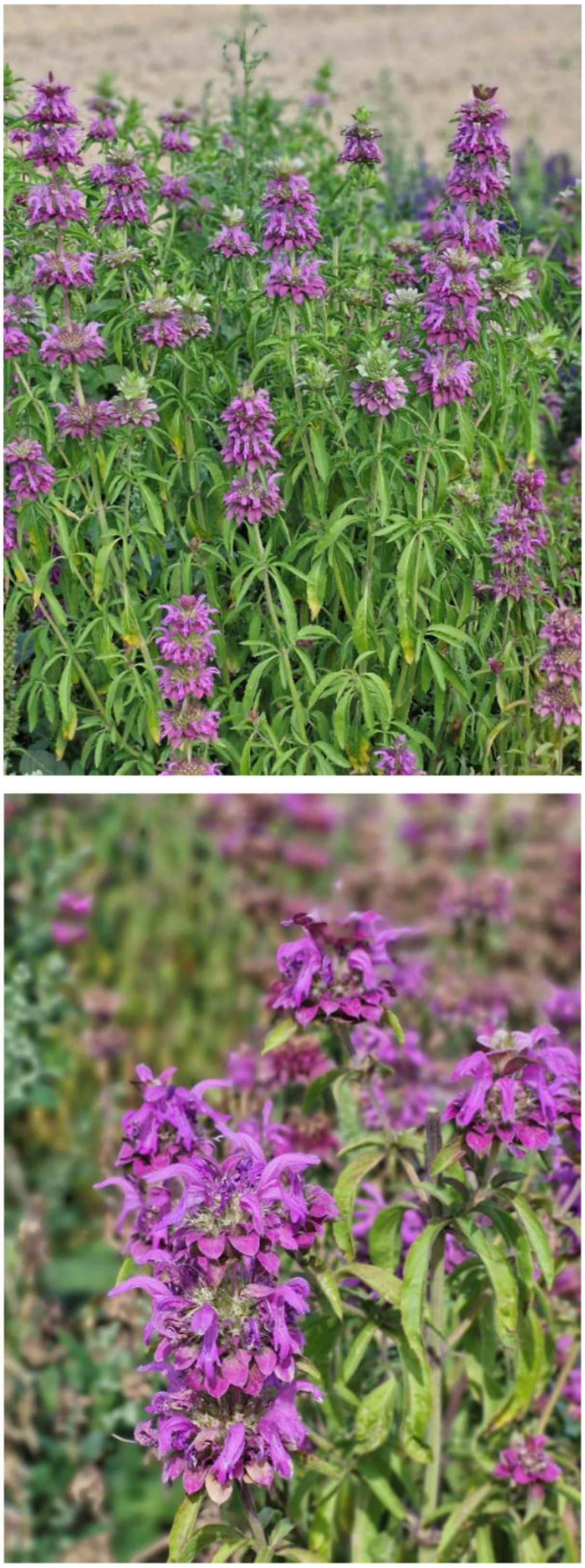

Figure S1. *M. didyma* plants used as the research material. The photograph shows plants in full flowering, when the flowers and above-ground parts were collected.
