# Supplementary material for: Chemical Composition, and Antioxidant and Antimicrobial Properties of Monarda didyma L.’s Essential Oils and Hydrosols
Source: Molecules. 2026 Jun 26;31(13):2252. doi: 10.3390/molecules31132252 (PMC13362633; doi:10.3390/molecules31132252)
Supplement: Supplementary file 1 [file molecules-31-02252-s001.zip › Table S1.pdf]

Table S1. The statistical reporting containing measures of variability (mean ± SD/SE).

Variable	Aggregated data						
	Mean	Min.	Max.	Variance	Standard deviation	Coefficient of variation	Standard Error.
Content (mL · 100 g^{-1} DM)	22.43	1.99	42.79	399.65	19.99	89.13	4.71
TPC mg GAE · mL^{-1}	188.43	5.76	427.62	36296.26	190.52	101.11	44.90
DPPH %	77.93	60.54	90.98	173.53	13.17	16.90	3.10
DPPH IC50 (μl)	0.200	0.001	0.440	0.042	0.206	103.079	0.049
FRAP mg Tr · g^{-1} DM	18.60	2.16	43.57	313.56	17.71	95.21	4.17
Variable	Essential oils data						
	Mean	Min.	Max.	Variance	Standard deviation	Coefficient of variation	Standard Error.
Content (mL · 100 g^{-1} DM)	3.01	1.99	3.79	0.36	0.60	19.90	0.20
TPC mg GAE · mL^{-1}	370.51	295.48	427.62	2528.24	50.28	13.57	16.76
DPPH %	90.26	89.58	90.98	0.18	0.43	0.47	0.14
DPPH IC50 (μl)	0.001	0.001	0.001	0.000	0.000	18.324	0.000
FRAP mg Tr · g^{-1} DM	34.32	19.83	43.57	110.09	10.49	30.57	3.50
Variable	Hydrosols data						
	Mean	Min.	Max.	Variance	Standard deviation	Coefficient of variation	Standard Error.
Content (mL · 100 g^{-1} DM)	41.85	41.15	42.79	0.30	0.54	1.30	0.18
TPC mg GAE · mL^{-1}	6.34	5.76	7.99	0.48	0.70	10.99	0.23
DPPH %	65.59	60.54	72.60	26.27	5.13	7.81	1.71
DPPH IC50 (μl)	0.399	0.334	0.440	0.001	0.033	8.209	0.011
FRAP mg Tr · g^{-1} DM	2.88	2.16	3.38	0.18	0.43	14.88	0.14
